# Food Insecurity Experiences Predict Children's Fruit and Vegetable Consumption in the USA

**DOI:** 10.5402/2013/426029

**Published:** 2013-06-19

**Authors:** Larry L. Howard

**Affiliations:** Department of Economics, California State University, Fullerton, 800 N. State College Boulevard, Fullerton, CA 92834-6848, USA

## Abstract

This research analyses the longitudinal relationships between household food insecurity (very low and low food security) experiences and children's consumption (servings/week) of fruit, green salad, carrots, potatoes, and other types of vegetables. Using a panel of 5,670 children aged 10–13 years who were first observed in spring 2004 and then again in spring 2007 at age 13–16 years, the main findings are as follows: first, children experiencing low food security consume significantly (*P* < 0.05) more fruit per week. In contrast, children experiencing very low food security consume significantly more carrots and potatoes per week, and estimates based on gender-stratified models indicate that the association is strongest among girls. Second, activity patterns are significantly related to children's dietary patterns; physical exercise is positively associated with fruit, green salad, carrot, and other vegetables consumption, while television watching is positively associated with potato consumption. Overall, the findings suggest that children living in food insecure home environments consume a greater number of servings of fruits and vegetables per week, relative to children living in food secure home environments.

## 1. Introduction

 A staggering number of families in the USA frequently restrict dietary intake because of financial hardship. Nearly eight million households with children (20.6% of the total) reported an inability to afford nutritionally adequate food in 2011, and over eight million children are currently food insecure [1]. Even though widespread hunger and malnutrition are uncommon, a healthful diet remains the most expensive alternative for families [2]. It has been recognized that nutrient deficiencies compromise children's growth and development [3–7] and that diet costs are positively related to fruit and vegetable intake [8]. In this study, we investigate how the intensity of food insecurity at home impacts children's weekly consumption of fruits and vegetables because households with limited financial means are more likely to adopt less expensive diets with low nutritional content [9]. 

From a policy perspective, it is necessary to assess whether food insecurity experiences reduce children's intake of items known to provide essential vitamins and minerals, particularly if dietary patterns persist [10, 11]. Insights into these relationships can help in evaluating the design of publicly funded programs aimed at preventing the undernourishment of children by targeting food sources that are inaccessible to households [12]. However, the cross-sectional evidence on differences in dietary intakes among children from food insecure households, relative to children from food secure households, has been mixed. For instance, Kirkpatrick and Tarasuk [13] analyze 24 h recall data and find that children aged 1–3 years from food insecure households consumed significantly fewer servings of fruits and vegetables. In contrast, they find no significant differences in fruit and vegetable consumption for boys and girls aged 9–13 years from food insecure households; and that boys aged 14–18 years consumed significantly less fruit and vegetables, but not girls in the same age group. Similarly, using food frequency questionnaire data, Pilgrim et al. [14] find that children aged 3 years from households with food insecurity consumed significantly less vegetables. Further, Lorson et al. [15] find no significant increase in the probability of lower daily fruit or vegetable intakes among food insecure children aged 2–18 years. While cross-sectional analyses are informative, a longitudinal analysis of children has the potential to afford stronger insights into how food insecurity impacts children's diets by controlling for potential confounding unobservable and difficult to measure characteristics related to dietary preferences of individuals and households. 

Households reporting food insecurity in the USA spend less on food relative to similar households with food security [1]. In light of the high obesity prevalence among children [16], a central concern is whether food insecurity stimulates a substitution of low-cost, energy-dense meals for healthy meals, which can increase the intensity of food insecurity within a household if children gain weight [17, 18]. The dietary behavior of children living in food insecure home environments may change for several reasons. Food insecurity measures used in developed countries assess both nutritional and psychological aspects of the household experiences [19, 20]. Children's diets can be impacted directly from the limited availability of food resources, but they can also depend on factors related to environmental stress. For instance, Weinreb et al. [21] emphasize the negative relationships between the mental well-being of parents and children and the experience of food insecurity, and Bhargava et al. [18] provide evidence that young children with parents who have worse emotional well-being and health limitations experience a greater intensity of food insecurity. Even though parents may attempt to protect children from direct hunger related stress by absorbing any food shortages themselves [22, 23], there remains the potential for household food insecurity to affect children's dietary behavior [24, 25].

The aim of this study is to examine whether household food insecurity experiences change the fruit and vegetable components of children's diets. Reductions in children's dietary intakes are certainly possible for financially constrained households with food insecurity. Yet, other adjustments in diets are also possible if only certain types of foods are unavailable to children. In this case, there are tradeoffs in consumption that may occur, both within and between food groups, for food insecure children with concurrent over- and undernutrition [26]. In the empirical analysis, we focus on the frequency of fruit and vegetable consumption because items in these food groups are important sources of essential vitamins and minerals that support growth and development through positive effects on immune function. 

## 2. Materials and Methods

### 2.1. Study Sample

The Early Childhood Longitudinal Study-Kindergarten (ECLS-K) began in the fall of 1998 by observing nearly 20,000 children in kindergarten enrolled in over 1,200 schools throughout the USA This study was conducted according to the guidelines laid down in the Declaration of Helsinki and all procedures involving human subjects/patients were approved by the ethics committees of the Department of Education and Office of Management and Budget, USA Written informed consent was obtained from all subjects/patients. Attrition due to geographical relocation resulted in approximately 9,000 children remaining in the study from kindergarten through 8th grade, and the locatable students were followed for a random 50% of the schools [27]. Our analytic sample consists of 5,670 children for whom full data were available at each time point in the spring of 2004 and 2007. Demographic characteristics of our analytic sample were similar to the full sample, and the available longitudinal sampling weights are utilized in the analysis to adjust for an oversampling of Asian and Pacific Islanders and nonresponse across the survey rounds. 

### 2.2. Fruit and Vegetable Consumption Measures

Children's consumption during the previous 7 days was assessed using food frequency questionnaires for fruit, green salad, carrots, potatoes, and other vegetables. The fruit category excludes fruit juice, and the potato category excludes fried potatoes or potato chips. Other vegetables consumed exclude green salad, carrots, and potatoes. The responses ranged from 1 to 7 corresponding to answers of none, 1–3 times, 4–6 times, 1 time per day, 2 times per day, 3 times per day, or 4 or more times per day, respectively. Using cell midpoints, outcomes are constructed to measure the number of servings consumed in the previous week and range from 0 to 28.

The food frequency questionnaire was first administered in spring 2004 and then again in spring 2007. For the initial round, an assessor read each question and recorded children's answers. In the subsequent round, children completed paper and pencil questionnaires themselves. The questions for fruit and vegetable consumption asked children to include any food eaten at home, school, restaurants, or other places during the previous week; and questions were based upon the Centers for Disease Control and Prevention/Division of Adolescent and School Health Surveys: the Youth Risk Behavior Surveillance Survey [27]. 

### 2.3. Food Insecurity Measures

Food insecurity levels in children's home environments in the previous twelve months were assessed using the United States Department of Agricultures (USDA) Household Food Security Survey Module. It consists of eighteen questions that are designed to capture information about the household environment such as anxiety over insufficient food budget or supply, perceptions of inadequate food quality or quantity, and instances of reduced food intake by household members; and each question specifies that the circumstance must have occurred in the past 12 months due to financial limitations [19]. The USDA considers households as food insecure if three or more questions are answered affirmatively, and households are further classified by increasing intensity as “low food security (3–7 affirmative responses)” or “very low food security (8–18 affirmative responses)” [1].

### 2.4. Control Measures

Extensive data were collected in the spring of each round using direct child assessments and parent interviews conducted by telephone [27]. The time-varying control variables measure child-specific and ecological factors influencing children's consumption of fruits and vegetables. Children's heights and body weights were measured in each round using a Shorr Board (Shorr Production, Olney, MD) and digital scale, respectively; duplicate measures were taken and we use the mean values to construct a Body Mass Index (BMI). BMI is typically compared against national standards based on age and gender to gain perspective on children's physiological development and overall nutritional status [28]. Minutes per day spent watching television and the frequency of physical activity lasting at least 20 minutes during the previous week were assessed by student questionnaires and are used to control for behavioral factors affecting children's diets, appetites, and metabolism [29, 30]. At the household level, controls include the number of siblings, the overall household size, a series of indicator variables corresponding to household income categories in US $1000 (<5, 5–10, 10–15, 15–20, 20–25, 25–30, 30–35, 35–40, 40–50, 50–75, 75–100, 100–200, and >200), and a series of indicator variables corresponding to parental education levels (8th grade or below, 9th–12th grade, high school diploma/GED, vocational program, some college, bachelor's degree, graduate/professional school with no degree, master's degree, and doctorate or professional degree). The correlation matrix is not reported here for brevity, however, the correlation between the control measures and the food insecurity measures is not higher than 0.17 for any one variable.

Several additional variables control for potential confounding effects of the local economic environment on household transitions between food security and food insecurity. Information on USA counties is linked to each child based on the location of the elementary school they attend. Control measures for characteristics of the local county economic environment during the previous year when food insecurity was assessed include average household income, annual unemployment rate, and the number of Food Stamp Program recipients per capita. Finally, an indicator variable for spring 2007 is included. 

### 2.5. Models

We investigate the relationships between food insecurity experiences and children's fruit and vegetable consumption using subject-specific regression models. The first model postulated for the total number of servings consumed in the previous 7 days for child *i* observed at time *t* is given in

(1) servingsit  =β0+β1  (food  insecurity)it   +β2  (BMI)it   +β3  (physical  exercise)it   +β4  (television  watching)it   +β5  (time-varying  covariates)it+ci+st+eit.

The time-invariant subject-specific correlation among repeated outcomes, *c*_i_, is explicitly modeled in the conditional mean [31], as well as the subject-invariant correlation among children's outcomes within a survey round, *s*_*t*_. This approach provides the least biased estimates of the effects of the explanatory variables by controlling for the influence of all unobservable and observed time-invariant factors affecting children's servings consumed during the previous 7 days. The estimated *β*_1_-coefficient indicates the difference in the number of servings for children from households that are experiencing food insecurity relative to children with food security. A limitation with the model in (1) is that the intensity of food insecurity experiences is assumed to have equal effects throughout the entire range of 3–18 affirmative responses on the USDA Household Food Security Survey Module. To further investigate potential exposure-response relationships in this context, we estimate the following regression model

(2) servingsit=β0+β1  (very  low  food  security)it+β2  (low  food  security)it+β3  (BMI)it+β4  (physical  exercise)it+β5  (television  watching)it+β6  (time-varying  covariates)it+ci+st+eit.

Very low food security is defined as 8–18 affirmative responses and low food security is defined as 3–7 affirmative responses on the USDA Household Food Security Survey Module.

### 2.6. Statistical Methods

Subject-specific linear regression methods are utilized to estimate the relationships between food insecurity experiences and children's fruit and vegetable consumption. The Stata *xtreg* (version 11, StataCorp LP) procedure is used in the estimation, and the cluster procedure is used to allow for an arbitrary pattern of correlation among repeated child observations. ECLS-K longitudinal sampling weights are utilized in all models to adjust for an oversampling of Asian and Pacific Islanders and nonresponse. Analyses are conducted using the full sample and gender-stratified samples. Differences are considered significant at the 5% level (*P* < 0.05).

## 3. Results

### 3.1. Descriptive Statistics

The sample means of the variables utilized in the models are reported in Table 1. Approximately 6-7% of children were from households experiencing food insecurity in any one time period. From spring 2004 to 2007, the number of children with very low food security at home increased from 1.7% to 1.9%, while the number of children with low food security at home decreased from 5.2% to 4.5%. The mean number of days per week children physically exercised for periods of at least 20 minutes significantly (*P* < 0.05) increased from 3.7 to 4.7, and the percentage reporting daily exercise increased from 11% to 25%. The mean number of minutes per day spent watching television significantly increased from approximately 140 to 213. Average education levels of parents were between 5 and 6 over this period, which correspond to having had “some college” or a “bachelor's degree,” respectively. Average household incomes over this period were between 9 and 10, which correspond to a range of $40,000–75,000.

### 3.2. Results for Children's Fruit and Vegetable Consumption

Table 2 presents the results from the subject-specific model in (1) for each of the five consumption outcomes. Food insecurity, defined as having either very low or low food security, is significantly associated with children's servings of fruit consumed during the previous week (*β*_1_ = 2.34, *P* < 0.005). Stratification by gender shows that the association between food insecurity experiences and children's fruit consumption is significant for boys (*β*_1_ = 2.55, *P* < 0.038) and girls (*β*_1_ = 2.09, *P* < 0.040); and two-tailed *t*-tests [32, 33] indicate the association is significantly (*P* < 0.001) different between boys and girls. In contrast, household food insecurity experiences are not significantly related to changes in children's consumption of green salad, carrots, potatoes, and other vegetables.

To investigate possible exposure-response relationships in this context, Table 3 presents the results from the subject-specific model in (2) for each of the five consumption outcomes. In this case, low food security experiences predict children's consumption of fruit (*β*_2_ = 2.33, *P* < 0.007); however, no significant differences are found for children with very low food security at home (*β*_1_ = 2.37, *P* < 0.153). Although the estimated coefficients are close in magnitude, the estimate for children with very low food security is relatively more imprecise. Further, children with very low food security consume significantly more servings of carrots (*β*_1_ = 1.50, *P* < 0.032) and potatoes (*β*_1_ = 2.39, *P* < 0.009) per week. Stratification by gender shows that the association between very low food security experiences and children's carrot and potato consumption is significant for girls, but not for boys. Both very low and low food security experiences remain insignificant predictors of green salad and other vegetables consumption.

Overall, children's activity patterns appear to explain part of the variation in children's consumption. Physical exercise is positively associated with the servings of fruit (*β*_4_ = 0.2108, *P* < 0.017), green salad (*β*_4_ = 0.1166, *P* < 0.012), carrots (*β*_4_ = 0.1265, *P* < 0.008), and other vegetables (*β*_4_ = 0.1856, *P* < 0.008) consumed in the previous week. Stratification by gender shows physical exercise is most strongly associated with green salad, carrot, and other vegetables consumption among girls, while for boys it is only significantly associated with fruit consumption. In contrast, television watching predicts potato consumption (*β*_5_ = 0.0013, *P* < 0.036) in the full sample, but not in gender-stratified samples, and it significantly predicts boys consumption of fruit (*β*_5_ = 0.0035, *P* < 0.030).

## 4. Discussion

Several insights are evident from the results. First, modeling the intensity of children's food insecurity experiences reveals important differences in their weekly consumption of fruit, carrots, and potatoes. While children with low food security at home consume a significantly higher number of servings of fruit, children with very low food security do not. Similarly, we find that children with very low food security at home consume a significantly higher number of servings of carrots and potatoes, and children with low food security do not. The USDA Center for Nutrition Policy and Promotion Food Prices Database for 2003-2004 contains average national prices for commonly consumed items in the USA There are twenty types of raw fruit sampled that cost less than $4 per pound; and the average cost is $1.82, with a range of $0.72 per pound for bananas to $3.83 per pound for pineapple. In contrast, raw carrots cost an average of $1.09 per pound, and raw white and sweet potatoes cost an average of $0.73 per pound, with a range of $0.38 per pound for white potatoes and $1.08 for sweet potatoes. Our findings suggest that a greater intensity of household food insecurity results in children, particularly girls, substituting relatively low-cost food items for more expensive food items. If households with very low food security are more financially constrained in comparison to households with low food security, the results are consistent with earlier findings showing that financial constraints affect food selection [9].

Second, the detailed information on children's activity patterns incorporated into our models provides an opportunity to assess the association that these behavioral factors have over time with dietary patterns. We find that children who exercise more frequently consume more fruit, green salad, carrots, and other vegetables, and that daily exercise is associated with consuming approximately 1 additional serving of each food type per week relative to children who do not exercise at all. Further, television watching is positively related to all children's consumption of potatoes. While the average effect is small in magnitude, nearly 13 hours of television watching per day corresponds to a one-serving increase in potato consumption, the relationship does suggest sedentary behavior is associated with an increase in energy-dense food. Public policies aimed at reducing the prevalence of obesity among children by augmenting their activity patterns may also have the additional benefit of shifting dietary patterns toward a more healthful diet.

## 5. Conclusions

Using within-subject (child/household) variation in reported food insecurity experiences for a panel of 5,670 children age 10–13 years who were first observed in spring 2004 and then again in spring 2007 at age 13–16 years, we estimate the effect of the experiences on the number of servings consumed per week of fruit, salad, carrots, potatoes, and other types of vegetables. The main findings indicate children with low food security consume a significantly higher number of servings of fruit, relative to children with very low food security or food security. In contrast, children with very low food security consume a significantly higher number of servings of carrots and potatoes, relative to children with low food security or food security. The pattern of dietary adjustments is consistent with increasing household financial limitations resulting in the availability of food items that are relatively less expensive nutritional sources. While children from food insecure households appear to increase the frequency with which they consume certain items, we do not find evidence that food insecurity experiences negatively impact children's diets in terms of the number of servings of fruits and vegetables consumed in a given week.

We caution against drawing stronger conclusions on the overall effect of food insecurity experiences on children's nourishment as there are important limitations of the empirical analysis presented here. First, the limited scope of the food frequency questionnaires used in the study prevents us from investigating other potential adjustments to children's diets in response to food insecurity. For example, we are unable to examine whether there are tradeoffs in children's consumption of grains or high-protein foods. Any nutritional benefits from increases in the consumption of fruits and vegetables may be offset by decreases in consumption of other foods. Second, we only observed children during the springtime and seasonal availability of fruits and vegetables may impact household dietary choices. Although we controlled for subject-invariant correlation among children's outcomes within a survey round, it is possible that food insecure households may respond differently to seasonal availability, as compared to food secure households. Third, the dietary outcomes we examine here only measure the number of servings consumed by children in the previous 7 days. A short time horizon of one week may be insufficient to accurately assess children's dietary habits and any persistent changes therein that are attributable to food insecurity. In conclusion, collecting more extensive data on children's diets would afford deeper insights into the extent to which households allocate food resources in response to the intensity of food insecurity experiences and the impact of such household-level changes on the dietary patterns of children.

## Figures and Tables

**Table 1:** Selected variables for children in the Early Childhood Longitudinal Study-Kindergarten observed in spring 2004 and 2007^a^. (Mean values and standard deviations for 5670 subjects).

Variable	2004		2007	
	Mean	SD	Mean	SD
Fruit servings per week (0–28)	7.53	7.76	7.21	6.77
Green salad servings per week (0–28)	2.18	3.82	2.54	3.54
Carrots servings per week (0–28)	2.94	5.38	1.90	3.37
Potatoes servings per week (0–28)	1.79	3.06	2.02	2.83
Other vegetables servings per week (0–28)	5.24	6.25	5.07	5.16
Very low food security (0-1)	0.017		0.019	
Low food security (0-1)	0.052		0.045	
BMI (kg/m^2^)	20.37	4.57	22.58	5.13
Physical exercise >20 min (d/week)	3.70	1.82	4.68	1.94
Watch television (min/d)	139.85	69.29	212.86	169.01
Siblings (*n*)	1.52	1.07	1.45	1.07
Household size (*n*)	4.52	1.24	4.44	1.26
Parental education level (1–9)	5.37	1.94	5.42	1.94
Household income category (1–13)	9.01	2.93	9.33	2.79
Boys (%)	48.50			
County-level characteristics^b^				
Unemployment rate (%)	6.06	2.03	4.69	1.19
Food stamp recipients (per capita)	0.08	0.05	0.09	0.06
Income ($ per capita)	32,105	9241	38,133	11681

^a^Very low food security is defined as 8–18 affirmative responses and low food security is defined as 3–7 affirmative responses on the USDA Household Food Security Survey Module.

^b^County-level unemployment rate data are from the U.S. Bureau of Labor Statistics. Food stamp recipient data are from the County-Level Food Stamp Recipient File, U.S. Bureau of the Census. County-level per capita income data are from the Regional Economic Information System, U.S. Department of Commerce, Bureau of Economic Analysis, Regional Economic Measurement Division.

**Table 2:** Estimates from subject-specific models of the effects of very low or low food security experiences on the number of servings of fruit and vegetables consumed by children during previous 7 days^a, b, c^. *β*-Coefficients and *P* values.

Outcome	*N*	Very low or low food security (0-1)	BMI (kg/m^2^)	Physical exercise (d/week)	Television watching (min/d)
Fruit					
All	5670	2.34 (0.005)	0.017 (0.854)	0.2107 (0.017)	0.0018 (0.153)
Boys	2750	2.55 (0.038)	0.211 (0.135)	0.2696 (0.038)	0.0035 (0.030)
Girls	2920	2.09 (0.040)	–0.144 (0.185)	0.1541 (0.141)	–0.0004 (0.827)
Green salad					
All	5670	0.64 (0.154)	0.031 (0.449)	0.1158 (0.012)	–0.0003 (0.675)
Boys	2750	0.76 (0.172)	0.053 (0.234)	0.0864 (0.176)	–0.0003 (0.710)
Girls	2920	0.67 (0.342)	0.001 (0.989)	0.1456 (0.031)	–0.0002 (0.746)
Carrots					
All	5670	0.65 (0.144)	0.047 (0.334)	0.1232 (0.009)	–0.0005 (0.386)
Boys	2750	1.05 (0.115)	0.191 (0.001)	0.065 (0.269)	–0.0006 (0.445)
Girls	2920	0.49 (0.397)	–0.092 (0.184)	0.1583 (0.034)	–0.0006 (0.477)
Potatoes					
All	5670	0.34 (0.500)	–0.025 (0.514)	–0.0003 (0.996)	0.0013 (0.037)
Boys	2750	0.05 (0.955)	0.003 (0.953)	0.0783 (0.390)	0.0013 (0.068)
Girls	2920	0.64 (0.165)	–0.051 (0.292)	–0.0779 (0.078)	0.0012 (0.287)
Other vegetables					
All	5670	0.26 (0.723)	0.015 (0.798)	0.1819 (0.010)	–0004 (0.630)
Boys	2750	0.17 (0.884)	0.069 (0.374)	0.0842 (0.406)	0.0012 (0.186)
Girls	2920	0.48 (0.586)	–0.064 (0.444)	0.2635 (0.007)	–0.0021 (0.180)

^a^Very low or low food security is defined as ≥3 affirmative response on the USDA Household Food Security Survey Module.

^b^ *P* values are adjusted for within-child correlation among repeated observations, and longitudinal ECLS-K sampling weights adjusted for oversampling and nonresponse.

^c^Controlling for number of siblings, household size, a series of household income category indicator variables, a series of parental education level indicator variables, an indicator variable for spring 2007, and county-level characteristics measuring income per capita, annual unemployment rate, and Food Stamp Program recipients per capita.

**Table 3:** Estimates from subject-specific models of the effects of very low and low food security experiences on the number of servings of fruit and vegetables consumed by children during previous 7 days^a, b, c^. *β*-Coefficients and *P* values.

Outcome	*N*	Very low food security (0-1)	Low food security (0-1)	BMI (kg/m^2^)	Physical exercise (d/week)	Television watching (min/d)
Fruit						
All	5670	2.37 (0.153)	2.33 (0.007)	0.017 (0.854)	0.2108 (0.017)	0.0018 (0.153)
Boys	2750	2.67 (0.309)	2.52 (0.041)	0.210 (0.133)	0.2700 (0.038)	0.0035 (0.030)
Girls	2920	1.62 (0.259)	2.22 (0.046)	−0.143 (0.188)	0.1519 (0.147)	−0.0004 (0.828)
Green salad						
All	5670	0.84 (0.088)	0.59 (0.234)	0.030 (0.458)	0.1166 (0.012)	−0.0003 (0.674)
Boys	2750	0.93 (0.186)	0.72 (0.221)	0.053 (0.238)	0.0869 (0.176)	−0.0003 (0.709)
Girls	2920	1.09 (0.136)	0.56 (0.481)	0.0001 (0.999)	0.1476 (0.029)	−0.0002 (0.744)
Carrots						
All	5670	1.50 (0.032)	0.43 (0.357)	0.045 (0.356)	0.1265 (0.008)	−0.0005 (0.384)
Boys	2750	1.52 (0.137)	0.93 (0.183)	0.190 (0.001)	0.0664 (0.256)	−0.0006 (0.446)
Girls	2920	1.71 (0.049)	0.17 (0.777)	−0.095 (0.172)	0.1639 (0.028)	−0.0006 (0.468)
Potatoes						
All	5670	2.39 (0.009)	−0.21 (0.712)	−0.030 (0.414)	0.0077 (0.877)	0.0013 (0.036)
Boys	2750	1.69 (0.195)	−0.39 (0.703)	−0.002 (0.967)	0.0837 (0.343)	0.0013 (0.067)
Girls	2920	3.08 (0.004)	0.01 (0.982)	−0.057 (0.242)	−0.0668 (0.134)	0.0011 (0.286)
Other vegetables						
All	5670	1.22 (0.287)	−0.0002 (0.999)	0.013 (0.830)	0.1856 (0.008)	−0.0004 (0.627)
Boys	2750	1.71 (0.303)	−0.24 (0.853)	0.064 (0.409)	0.0892 (0.368)	0.0012 (0.184)
Girls	2920	0.76 (0.560)	0.41 (0.671)	−0.064 (0.440)	0.2648 (0.007)	−0.0021 (0.179)

^a^Very low food security is defined as 8–18 affirmative responses and low food security is defined as 3–7 affirmative responses on the USDA Household Food Security Survey Module.

^b^ *P* values are adjusted for within-child correlation among repeated observations, and longitudinal ECLS-K sampling weights adjusted for oversampling and nonresponse.

^c^Controlling for number of siblings, household size, a series of household income category indicator variables, a series of parental education level indicator variables, an indicator variable for spring 2007, and county-level characteristics measuring income per capita, annual unemployment rate, and Food Stamp Program recipients per capita.
